# Autonomous and Assisted Control for Synthetic Microbiology

**DOI:** 10.3390/ijms21239223

**Published:** 2020-12-03

**Authors:** Alvaro Banderas, Matthias Le Bec, Céline Cordier, Pascal Hersen

**Affiliations:** 1 Institut Curie, Université PSL, CNRS UMR168, Sorbonne Université, Laboratoire Physico Chimie Curie, 75005 Paris, France; matthias.lebec@curie.fr (M.L.B.); celine.cordier@curie.fr (C.C.); 2 Laboratoire MSC, UMR7057, Université de Paris—CNRS, 75013 Paris, France

**Keywords:** robustness, cybergenetics, relative sensing, microbial consortia, synthetic biology, control

## Abstract

The control of microbes and microbial consortia to achieve specific functions requires synthetic circuits that can reliably cope with internal and external perturbations. Circuits that naturally evolved to regulate biological functions are frequently robust to alterations in their parameters. As the complexity of synthetic circuits increases, synthetic biologists need to implement such robust control “by design”. This is especially true for intercellular signaling circuits for synthetic consortia, where robustness is highly desirable, but its mechanisms remain unclear. Cybergenetics, the interface between synthetic biology and control theory, offers two approaches to this challenge: external (computer-aided) and internal (autonomous) control. Here, we review natural and synthetic microbial systems with robustness, and outline experimental approaches to implement such robust control in microbial consortia through population-level cybergenetics. We propose that harnessing natural intercellular circuit topologies with robust evolved functions can help to achieve similar robust control in synthetic intercellular circuits. A “hybrid biology” approach, where robust synthetic microbes interact with natural consortia and—additionally—with external computers, could become a useful tool for health and environmental applications.

## 1. Introduction

Homeostasis is the ability to maintain physiological parameters at steady levels, for example, body temperature or blood salt concentration in an organism and turgor pressure or macromolecular crowding in cells [1,2]. The robustness of the underlying molecular networks is a crucial component of cellular homeostasis. Robustness can be generally defined as the property that allows a system to maintain its functions, at least partially, in the presence of internal and external perturbations [3]. Robustness has been observed in a variety of molecular systems, including the pathways that control gene expression, metabolism and cellular signaling [4], with negative feedback being at the core of the operation of such circuits. Advances towards a quantitative definition of biological robustness have emerged from the similarity between negative feedback in electronic circuits and negative autoregulation in genetic circuits [5]. Electronic and biological circuits can both be seen as information processing flows and share conceptual similarities regarding the description of their dynamics and sensitivity to external perturbations. For both, one can define the robustness of a circuit based on the ratio of the relative change in steady-state output to the relative change in each parameter value.

Synthetic biology aims to construct genetic circuits from the bottom-up for both applied and fundamental research. However, the predictability and scalability of synthetic circuits remain poor overall [6], making implementation of robust (and therefore reliable) circuitry desirable. Synthetic biologists have already proposed theoretically and produced experimentally such robust genetic circuits. These advances pave the way towards the construction of more complex cellular networks with predictable and useful functions, which could enable desired complex cellular behaviors to be engineered from the bottom-up.

Systematic, quantitative characterization of the wide range of uncertainties that affect synthetic circuits, as well as the variety of access points to spatiotemporally and orthogonally control natural circuits, has been reviewed elsewhere [6,7]. Here, we focus on how nature-inspired circuitry can provide design schemes and components to accomplish robust control over both synthetic and natural complex cellular ecologies. First, we introduce robust control in natural intra- and intercellular circuits, focusing on examples with well-described derived biological functions. Second, we establish the challenges involved in applying such knowledge to the problem of controlling synthetic ecologies. Third, we introduce cybergenetics as a multifaceted solution, and divide the approach into external (computer-aided) and internal (autonomous) interventions. Finally, we explore how such interventions may help to achieve robust control of natural populations for biomedical applications.

## 2. Natural Robust Control

### 2.1. Perfect Adaptation and Relative Sensing of Stimuli

Robust perfect adaptation—where large external perturbations are attenuated back to a baseline—is a feature that can be useful to maintain outputs at desired levels in synthetic systems. Furthermore, it could help to generate biologically and ecologically relevant input–output response patterns for synthetic microbes, as it does in their natural counterparts. Bacterial chemotaxis is a prototypical example of natural robust control leading to a relevant biological function. Robust perfect adaptation is achieved in *Escherichia coli* via an integral negative feedback strategy [8,9,10], where the output is integrated over a period of time before being fed back to the input. When an *E. coli* cell senses an increase in the nutrient concentration as it explores the environment, its chemoreceptors become less sensitive, allowing cells to sense nutrients across a wide range of concentrations without saturating their response, while also reducing variability (noise) among responding cells. This mechanism results in response magnitudes that follow the Weber–Fechner law for sensory systems (or logarithmic sensing) [11,12,13], where the perceived magnitude (a pathway output in the case of cells) is proportional to the logarithm of the input magnitude. In practice, chemotactic responses of *E. coli* are then proportional to the fractional gradient (gradient normalized to the chemoattractant concentration) of nutrients—rather than the absolute gradient [14,15,16], allowing *E. coli* to climb up exponential gradients with constant drift velocity. Similar relative sensing strategies have been observed in a variety of cellular and biochemical systems with different underlying mechanisms, notably the incoherent feed-forward loop (IFFL) [11,17,18,19,20], showing that sensing relative stimuli is of primary importance to a cell’s performance in its native environment, and that mechanisms other than integral feedback can bring about perfectly adapting relative stimulus sensing.

Natural control topologies, such as the one in *E. coli* chemotaxis, can provide inspiration for the design of circuits with complex behavior. Implementing similar capabilities in synthetic microbes might therefore be useful for achieving improved performance in complex ecosystems.

### 2.2. Sensing Relative Population Composition

**Figure 1:**
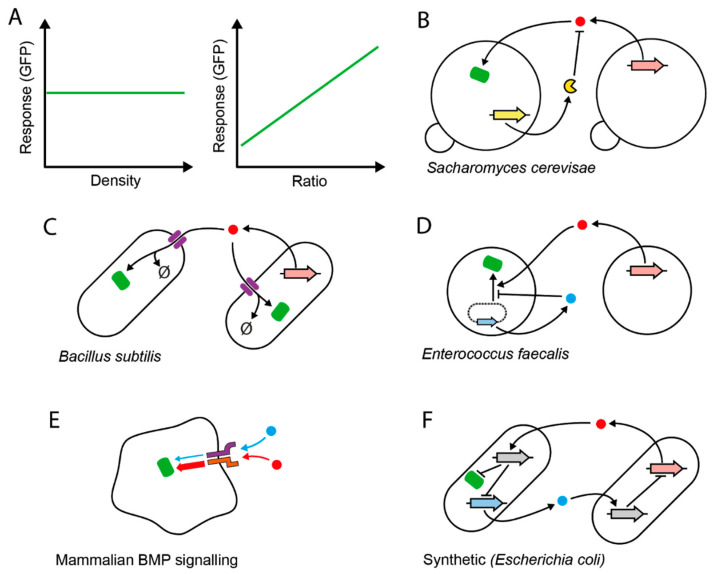
Relative sensing of population parameters. (**A**) Theoretical perfect ratio sensing. The mean gene expression output of a reporter population (Response) is insensitive to changes in the total density of the co-culture, but sensitive to the relative abundance of the individual populations. (**B**–**D**) Natural microbial intercellular signaling networks composed of distinct cell populations that perform ratio sensing. A stimulatory signal (red circle) accumulates in the media in proportion to the density of the signal emitter and stimulates green fluorescent protein (GFP) production (green). The concentration of the signal also depends on antagonistic activity, which balances out activation. For this, *S. cerevisiae* (**B**) uses an extracellular protease (yellow), which directly degrades the signal produced by partner cells. *B. subtilis* (**C**) depletes the signal by internalizing it through active pumps (purple) and degrading it (∅ symbol) internally. In *Enterococcus faecalis* (**D**)**,** cells carrying a conjugative plasmid (dotted line) produce an inhibitory signal (blue circle) from a plasmidial gene (blue arrow), which antagonizes the interaction between the signal produced by plasmid-free cells (red) and its cognate transcription factor (not shown). The thick arrows correspond to the genes encoding the corresponding products. (**E**) Ratiometric sensing of distinct extracellular signals in mammalian cells. One of the two signals (blue) forms receptor–signal complexes with low activity, while the other (red) forms high-activity complexes, such that one signal (blue) competitively inhibits activation by the other stronger ligand (red). Receptors are represented by the purple and orange symbols. Thick and thin arrows pointing to GFP (green) represent high and low activity of the receptor complexes, respectively. (**F**) Synthetic intercellular toggle switch. Signals (blue and red) produced by each cell from their respective genes (thick blue and red arrows, respectively) inhibit the production of signals by the other cell in a co-repressive circuit, via signal-specific expression of a transcriptional repressor (its coding gene is shown in grey). In this case, the per-cell output level is directly proportional to the ratio of the blue signal-producing strain to the red signal-producing strain (“majority wins”). The opposite pattern (“minority wins”) can be obtained by changing the circuit such that GFP is directly inducible by the red signal instead of repressed by the induced transcription factor (grey).

Another form of relative sensing—in this case pertaining to the population level—is ratiometric sensing, for which a handful of examples have been described (Figure 1). Ratio sensing is the ability of cells to produce an output proportional to the composition of the cell population, with the output remaining robust to variations in total cell density (Figure 1A). One example is the mating-pheromone pathway of *Saccharomyces cerevisiae* [21] (Figure 1B). During mating, two mating types communicate via extracellular pheromone signals to activate mating responses such as cell–cell agglutination and cell-cycle arrest. Mating-type *MAT*α produces the α-factor pheromone, which activates *MAT*a cells in proportion to the concentration of *MAT*α. However, *MAT*a also produces the extracellular protease Bar1, which degrades α-factor at a rate proportional to the concentration of *MAT*a. This system enables *MAT*a cells to remain sensitive to ratio changes and be less sensitive to the total population density. Another example is the PhrA-RapA-Spo0F signaling pathway in *Bacillus subtilis* (Figure 1C). When part of the population “cheats” by not producing an extracellular signal that benefits the population [22], the ratio of producers to “cheaters” can specifically be sensed by cells. This happens because of a population-wide signal internalization or “pumping in” through a signal-specific permease and subsequent signal degradation. Again, equal increases in both cell densities increase production, but also increase the depletion rate—making the concentration of available signal proportional to the cell ratio. Similar ratio sensitivity can be found in plasmid conjugation in Gram-positive *Enterococcus faecalis* (Figure 1D)*,* where two antagonistic signals—each produced specifically by plasmid-carrying or plasmid-free cells—provide the necessary balance to maintain responses that are roughly insensitive to the total cell density [23,24]. Functionally, the yeast and *E. faecalis* systems regulate costly mating induction. As the ratio is a proxy for the likelihood of a successful random encounter with a mating partner, measuring it and acting accordingly avoids unproductive activation when mating chances are low. In a more distant example, the mammalian bone morphogenetic protein (BMP) signaling pathway can specifically compute the ratio of two particular BMP ligands (Figure 1E). This capacity directly arises from competitive receptor–ligand interactions [25]. The signals could potentially be produced by two specific cell types, and the circuit’s output could therefore report their ratio. Finally, a synthetic intercellular toggle-switch system can also function as a ratiometric sensing circuit [26] (Figure 1F). The system is designed to switch on or off depending on which cell type is in the majority. The output is effectively linearly dependent on the cell fraction for defined periods of time, making this system a potentially useful ratio sensor.

These systems share the general need for opposing activities, and do not necessarily require feedback motifs in their circuitry for their basic operation. Importantly for synthetic biology, these circuit topologies can potentially allow a two-cell population to inform a downstream process about which cell type is in the majority, and with varying degrees of precision, the current fraction of cells. Conversely, if linked to expression of growth-determining genes, this system could enable control of the ratio itself, with applications in microbial consortia with precise cellular-stoichiometry needs.

## 3. Synthetic Population Control

Natural microbial consortia exemplify how multi-organismic communities achieve robustness to environmental fluctuations by augmenting metabolic capacity through division of labor, and have inspired recent research to rationally design synthetic microbial consortia [27]. Similarly, synthetic consortia can be engineered to distribute the cost of heterologous expression of metabolic pathways, compartmentalize competing cross-inhibiting yet complementary pathways, and expand their metabolic capabilities compared to monocultures [28]. One potential application for synthetic sensors of relative population composition is to help create synthetic microbial consortia with a stable cellular composition. This is important for any process where the stoichiometry of the different activities performed by corresponding cells is determinant for optimal performance of the whole. However, current approaches generally suffer from long-term instability, as competition for resources and exponential growth drive the composition out of balance [29]. Recent attempts using cell lysis to control binary populations demonstrated higher stability [30]—yet can result in undesired proteins and other components in the spent cultures, which crucially limits the purity of the end-products for bioproduction applications. Moreover, this approach only works with highly biased initial cell ratios, as one population quickly overtakes the other when added at equal initial densities. Furthermore, much biomass (and therefore energy) is sacrificed for the benefit of population stability.

We argue that a microbial consortium with autonomous, robust control of cellular stoichiometry—a *ratiostat*—could be constructed, at least in its simplest form (two-cell population), by with two different strategies. First, synthetic circuits inspired by the ratiometers (Figure 1) could be constructed. This is increasingly possible thanks to the availability of several orthogonal (non-crosstalking) communication channels for synthetic biology, where up to six separate orthogonal channels have been implemented at one in bacteria [31,32,33,34]. Since the ratiometer’s readout ignores fluctuations in total cell density and provides precise composition measures for downstream processes, linking such output to cellular growth actuators provides an opportunity to attain stable compositions because responsive cells could induce growth according to current ratio measurements, e.g., if their fraction drops below a certain value. Second, there is also the possibility to build *ratiostats* through external control, by delocalizing the circuit complexity within a computer/algorithm and directly control cell growth. The advantage of computers is the possibility to control the composition of communities with several members more easily, as a single input to directly control growth in each population is needed. Both these strategies can be implemented using the cybergenetics framework, which brings useful tools from control theory to guide the design of the synthetic circuits.

## 4. Cybergenetic Control

Cybergenetics, the interface between synthetic biology and control theory, enables different types of control strategies, including external and internal control of biological circuits [35,36,37]. External control aims to regulate cell cultures, single cells, or complex cell assemblies through computer-assisted feedback. Specifically, information collected on a particular cellular state or states (e.g., a fluorescent protein that reports a signaling pathway output) is used to compute an appropriate intervention through chemical or physical inputs that change the cell to a new desired state in real-time. The computer measures these outputs dynamically and makes decisions on the timing and intensity of subsequent inputs; these decisions are dictated by a control algorithm that can vary in complexity. On the other hand, internal control uses DNA-encoded small regulatory networks containing feedback structures for a similar purpose. Although cybergenetics has not been explored extensively as a means of control and design intercellular circuits, its current use to control intracellular circuits can serve as guidance for such purpose. Thus, here we show some available examples. Cybergenetics has increasingly been used as a strategy to apply automated dynamic control for bioproduction [38,39], in this section however, we rather focus on the fundamental aspects of control; namely, how cybergenetics provides insight into natural biological behaviors and the initial steps required to control complex functions in synthetic systems.

### 4.1. External (Computer-Aided) Control

**Figure 2:**
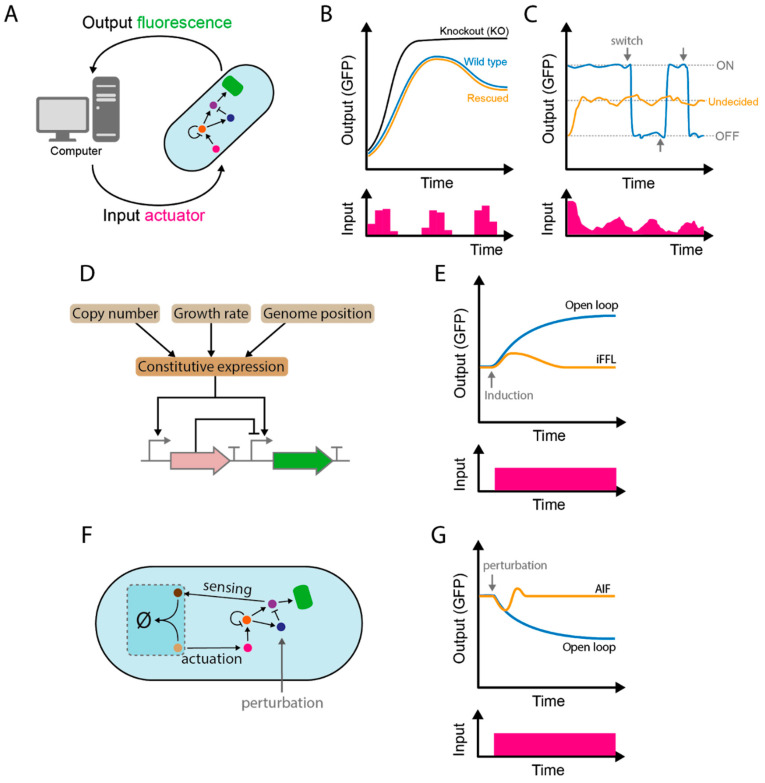
Internal and external control strategies from cybergenetics. (**A**) Computer-aided control. A network of interacting molecular components (circles) with a fluorescent protein output (green) interacts with a computer, which measures and acts on the output by delivering network inputs. (**B**) Dynamic compensation. Native (blue) and negative-feedback knockout (black) outputs for a signaling pathway, compared to computer-controlled negative feedback expression (orange). In this case, regular input pulses (bottom) restored wild-type behavior. (**C**) Application of external control to maintain a bistable system—in this case, a synthetic toggle switch circuit—close to its unstable equilibrium point. The circuit is switched on or off (blue) using two specific chemicals (arrows). By maintaining one input at roughly constant levels (not shown) and adding the other periodically (bottom), the system is maintained at its unstable point and remains undecided (orange). (**D**) Incoherent feed-forward loop (IFFL) based robust constitutive expression. Constitutive expression machinery activates the target gene (green, GFP) and its repressor gene (pink, transcription-activator-like effector (TALE) protein); the repressor gene is encoded upstream in the cassette and binds non-cooperatively to the target gene. Promoters and terminators are represented by right-angled and T-shaped lines, respectively. Sources of perturbations in the capacity for constitutive expression are shown in the upper three boxes. (**E**) Adaptation of output levels to an induced step increase (bottom) in the copy number of the incoherent feed-forward loop cassette (IFFL, orange) and a regular non-feedback system (blue). (**F**) A biomolecular (embedded) antithetic integral feedback controller based on inactivation (∅) by molecular titration (darker blue square, see text) controls the network, which reports a fluorescent output (as in A). The setpoint is determined by the ratio of the titrated elements (brown and beige circles). (**G**) Robust perfect adaptation of the antithetic integral feedback controller (AIF). Normally, a perturbation (a step function that activates degradation of circuit component; bottom) brings the system to a new steady state (blue). Using the control loop, the system is brought back to its set point (orange).

Computer-assisted feedback control (Figure 2A) provides an experimental platform to interrogate biological systems in unprecedented ways. Dynamic compensation represents one example of the cybergenetic approach to this issue [40] (Figure 2B). To understand the role of feedback regulatory elements in biological signal transduction, biologists have traditionally relied on gene-knockouts and genetic complementation. Dynamic compensation allows such complementation to be dynamically modulated in real-time. This approach was used to explore the roles of the various negative feedbacks that act on upstream elements of a prototypical MAPK signaling pathway in *S. cerevisiae*. By optogenetically inducing various elements via a real-time control loop, the authors revealed the dynamic requirements that such feedback processes must possess to preserve wild-type function. These requirements varied depending on the element studied; for example, the phosphatase Msg5 had to be provided in pulses to recover wild-type function, whereas the negative regulator of G protein signaling Sst2 did not have any dynamic requirements, and a constant step input sufficed. Similar approaches have yielded insight into transcriptional dynamics using spatiotemporal delivery of inputs [41], spatiotemporal control of gene expression in multiple single cells [42] and virtual pattern formation [43].

External control has also enabled the exploration of states normally not maintained by cells for long; for example, maintaining a bistable molecular circuit in its unstable transition state. Lugagne et al. [44] used a synthetic toggle switch in *E. coli* to demonstrate the feasibility of this approach (Figure 2C). The system has two stable equilibrium points that can be switched by the addition of specific chemical signals, and one unstable equilibrium point corresponding to an “undecided” state. Using these inputs and by following the behavior of single cells in a microfluidic device, the system could be periodically forced to maintain the undecided state. This approach provided a proof-of-principle that could allow, for example, the study of transient cellular states such as cell differentiation or malignant transformation at high sample sizes for quantitative analyses. Further theoretical developments in this area include an improved robust version of periodic forcing through integral feedback [45] and ratio control [46], where rather than keeping a toggle switch undecided, computer feedback controls the proportions of cells in each of the two states.

### 4.2. Internal Cybergenetic Control

Internal control relies on the addition of control structures to synthetic circuits. Implementation of robust perfect adaptation (Section 2.1) is one way to increase the performance of synthetic circuits through internal control. For example, IFFLs [47,48] have been used to ensure constant levels of chromosome-inserted circuit components that are otherwise highly susceptible to variation in the genetic context or variation in genetic dosages due to multiple insertions (Figure 2D,E). In one example [49], the authors used an IFFL strategy based on compensatory non-cooperative transcriptional inhibition provided by transcription-activator-like effectors (TALEs). Similarly, synthetic circuits that combine an IFFL with negative feedback in mammalian cells enable robust gene expression against gene dosage variations [49].

Antithetic integral feedback [50,51] is another circuit that allows perfect adaptation (Figure 2D). The antithetic integral feedback circuit is a synthetic biological implementation (in *E. coli*) of a generalized model born from control theory, but nevertheless present in natural signaling pathways [8]. Rigorous mathematical treatment of robust perfect adaptation mediated by antithetic integral feedback shows that, if the technical challenges of implementation can be overcome, this strategy can eventually keep any cellular output of choice at steady levels. The feedback can be embedded in any arbitrary intracellular network with noisy dynamics, and the user-defined setpoint for the output will remain robust to a broad range of biochemical parameter values. Implementation of the antithetic control strategy crucially relies on proteins that stoichiometrically inactivate each other, such as those already proven useful in synthetic circuits [52,53]. In the antithetic controller, such molecular titration is achieved using the SigW/RsiW σ-factor/anti-σ-factor system from *Bacillus subtili*s [54,55]. The σ-factor SigW, which determines the output of the circuit (a fluorescent protein), is inactivated by the anti-σ-factor RsiW. The levels of SigW are determined by chemical activation of its constitutively expressed transcription factor. On the other hand, the inactivating counterpart is expressed in proportion to the levels of its own specific transcription factor, which can also be chemically activated. As both transcriptional regulators can be independently tuned, and output levels rely on the stoichiometry of the titrated partners (available free SigW), a setpoint for the output can be determined externally.

Proposed extensions of the antithetic integral feedback controller include a biomolecular proportional-integral-derivative controller, also inspired by control theory [56], and a cellular (rather than embedded) antithetic feedback controller composed of two cell types controlling the state of a third cell type [57]. Interestingly, an integral feedback controller has also been constructed in vitro, opening yet another avenue of cell-free regulation in synthetic biology [58]. Moreover, antithetic control was shown to ensure optimal biofuel production without knowledge of the system’s parameters [59].

## 5. Interactions between Controllers and Natural Populations

**Figure 3:**
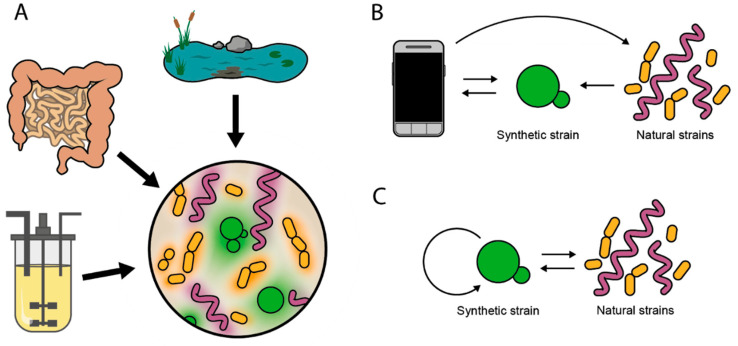
Ecosystem intervention. (**A**) Various ecosystems such as a natural water source, the human gut, or a bioreactor are susceptible to interventions using engineered cell populations to enable, e.g., remediation, therapy, or optimization processes, respectively. The engineered cells (green) coexist and interact with the natural microbiota (purple and yellow) via secreted molecules (corresponding colored halos around cells). (**B**) External control. Synthetic cells (center) can detect and report specific properties of the natural population (right). Data collection and computer-aided analysis (left) can be used to modify the detector itself (e.g., replenish the detector strain to avoid its extinction) or the natural population (e.g., add a specific dose of a species-specific toxin). (**C**) Internal control. The synthetic cell interacts bidirectionally with the natural microbiota. Using the information collected, the synthetic cell can both control itself (e.g., maintain its relative abundance) and the natural system (e.g., secrete killing agents).

The robustness strategies outlined above could be implemented in both intra and intercellular circuits for the improved robustness (and performance) of synthetic microbes used to directly intervene natural ecosystems (Figure 3A). For example, therapeutic microbes promise to provide innovative means for diagnosing or treating infection, cancer and other diseases using cells reprogrammed to perform specific functions. Important advances have been made in this field; for example, detection of cancer in mice using orally consumed reporter bacteria that produce an easy-to-read colorimetric output in urine [60] or several systems for drug delivery using bacterial lysis, e.g., to release nanobodies in the tumor microenvironment—inducing tumor regression in mice [61]—to synchronize cyclic delivery of drugs [62], and to kill a human pathogen using a species-specific antibiotic [63]. These microbes must sense and respond to dynamically changing environments and noisy signals, and therefore could benefit from robust circuit design. One way of improving the performance of microbes with engineered behaviors is to use external computer control to monitor and aid their stability and action, namely, by detecting properties of target cells, synthetic microbes provide externally measurable outputs that in turn guide external intervention, e.g., adding more sensors or a specific drug (Figure 3B). Here we rather focus on engineering microbes capable of performing tasks autonomously, i.e., of displaying specific engineered behaviors by robust intra- and intercellular circuit design only.

Synthetic pathogen-seekers are an interesting case of intracellular circuit susceptible to improvements by robust design. In one example [64], bacteria effectively followed gradients via a cleverly intervened chemotaxis system, where inputs enter the network transcriptionally—by inducing expression of the key phosphatase CheZ—rather than interacting with specific receptors, as natural chemoattractants do. Interestingly, this shows that an arbitrary signaling pathway with a transcriptional output could enable a sensed extracellular molecule to function as a chemoattractant, widening the spectrum of possibilities for engineering chemotaxis towards unnatural substrates, beyond the use of chemotaxis receptor engineering. Although promising, the narrow range of concentrations in which the protein CheZ must operate in the synthetic system [64] limits its performance. This provides an opportunity for robust control strategies to ensure its expression remains constant at the desired basal levels by, for example, ensuring perfect adaptation of CheZ levels through co-induced (as the case in Figure 2D,E)—rather than constitutive (as in the cited work)— antagonistic action (CheZ degradation). This could eventually bring engineered chemotaxis closer to wild type performance.

Intercellular robust circuits in the context of therapeutic microbes are a more exploratory idea. For example, engineered robustness could improve “controller cells” that interact with native populations in their ecosystem. Controller cells could potentially sense chemical cues produced naturally by microbial communities—which has become increasingly possible through engineered metabolite sensing [65]—and respond to achieve or restore specific states in the target population (e.g., specific pathogen killing) (Figure 3C). For such purpose, controller cells must first prevent their own extinction, ensure that their action only happens when in the majority (e.g., for effective killing) and activate suicide (e.g., for clearance). A controller-cell population—adapted to its target environment (as in [66])— equipped with ratiometric capacities (Section 2.2) could help to achieve the three tasks, by preventing its extinction by activating fast growth when in the minority, and activating lysis to release a specific toxin when in the majority.

## 6. Conclusions

Natural biochemical robust signaling networks keep constancy in the levels of specific outputs, with such levels being naturally selected in their native operating environments. Molecular sensory pathways integrate the information provided by signals that transiently alter those levels, to generate biologically meaningful responses. Therefore, the construction of synthetic cells that interact with complex ecosystems might benefit from the implementation of similar robust computational capabilities. With the limited amount of adequate signals that synthetic microbes can be engineered to sense in such scenario, the specific computation made is crucial, as signals can inform various relevant parameters of the physical and “social” environment [67,68]. In this sense, the relative sensing strategies presented, provide useful inspiration for designing circuits for effective interactions between synthetic cells and natural ecosystems. Making such interactions functional and safe in natural scenarios is however a difficult task. The advances presented here suggest that a way forward is to, starting from a niche-adapted chassis and robust circuit design, place computer algorithms in the control loop and force the microbe’s correct performance. By understanding the computer feedbacks necessary for such semi-autonomous display, we might in turn understand what is needed for synthetic circuits to improve both the cell’s autonomous behavior and capacity to die when necessary.
